# Dehumanization of Hospitalized Patients and Self-Dehumanization by Health Professionals and the General Population in Greece

**DOI:** 10.7759/cureus.20182

**Published:** 2021-12-05

**Authors:** Dimitra Lekka, Clive Richardson, Anna Madoglou, Konstantina Orlandou, Vassia I Karamanoli, Aikaterini Roubi, Christos Pezirkianidis, Vasileia Arachoviti, Athanasios Tsaraklis, Anastasios Stalikas

**Affiliations:** 1 Department of Psychiatry, Sotiria Thoracic Diseases Hospital of Athens, Athens, GRC; 2 Department of Economic and Regional Development, Panteion University of Social and Political Sciences, Athens, GRC; 3 Department of Psychology, Panteion University of Social and Political Sciences, Athens, GRC; 4 Psychology, Hellenic Military Academy, Athens, GRC; 5 Department of Dermatology, Sotiria Thoracic Diseases Hospital of Athens, Athens, GRC

**Keywords:** greece, patient, hospital, self-dehumanization, dehumanization

## Abstract

Introduction

Dehumanization is defined as the denial to people of their humanness. It is distinguished into animalistic and mechanistic dehumanization. The aim of this study is to examine whether professionals in a public hospital dehumanize the patient and self-dehumanize.

Methods

We used the Dehumanization Questionnaire, the Mechanistic Self-Dehumanization Scale, the Human Nature and Human Uniqueness Characteristics Questionnaire, the General Causality Orientation Scale and the Adult Attachment Questionnaire. The sample consisted of 135 mental health professionals (20 from a general hospital and 115 from a psychiatric hospital), 134 other health professionals from the general hospital and 84 people from the general population.

Results

Health professionals dehumanize the hospitalized patient more than the general population. The secure attachment acts protectively on self-dehumanization and negatively on the dehumanization of the hospitalized patient. Finally, autonomous people are not self-dehumanized.

Conclusions

Our findings indicate that measures should be taken for health professionals so that they do not dehumanize the patient.

## Introduction

The phenomenon of dehumanization - defined as the denial to people of their humanness [1] - goes hand in hand with human history. It has two different aspects, one referring to qualities that are unique to the human species (Human Uniqueness) and the other to qualities that are essential or fundamental to humans (Human Nature). Human Uniqueness characteristics, in addition to secondary emotions, include cognitive skills, intelligence, ethics: in short, cognitive complexity, culture, perfection, socialization, and intrinsic moral sensitivity [1,2]. The characteristics of Human Nature include qualities that are typically, radically or essentially human, comprising those characteristics that form the core of the concept of "human", connect man with the natural world and bodily biological aspects, appear early, prevail in populations and are universal in different cultures, that is, they are fundamental, inherent and natural [1,3].

Haslam's model of dehumanization is based on the absence of the two senses of humanity, where the two forms of dehumanization arise [1,4]. When human uniqueness characteristics such as kindness and moral sensitivity are not recognized in groups or in individuals, they are considered uncultured, coarse, without self-control, incomprehensible or irrational, childish, immature. Their behavior is guided by motivations, appetites and instincts without cognitive mediation [1,3,5,6]. This is the animalistic form of dehumanization, at the core of which is an obvious or tacit resemblance of humans to animals and highlights their beastly or barbaric features.

The denial of attributes of Human Nature to others results in the deprivation of emotionality, warmth, cognitive openness and individual agency. Thus the lack of emotion and warmth makes them inactive and cold, the lack of cognitive openness (e.g. curiosity, flexibility) makes them rigid and the lack of agency (agents are goal-directed entities that are able to monitor their environment to select and perform efficient means-ends actions) makes them passive and their behavior is automatically provoked and not promoted by personal will.

This combination of attributes leads to viewing others as objects or automatons. This form of dehumanization can therefore be characterized as mechanistic. Mechanistic dehumanization can make others unworthy of moral concern and care [7,8]. Dehumanization concerns not only perceptions of others but also perceptions of oneself. This may be the result of harmful treatment by others, or it may be caused by one’s own harmful behavior [9].

Self-dehumanization also has consequences for emotions and behavior. Self-dehumanization is associated with deterrent self-awareness, cognitive degenerative states, and feelings of shame, guilt, sadness, and anger. Self-dehumanization can also motivate behavior aimed at recovery, perhaps in an attempt to regain the humanness lost [10].

The purpose of this study is to link the concepts of dehumanization and self-dehumanization with self-determination theory and attachment theory, so that measures can be taken to prevent them and improve the quality of health care that promotes patients' quality of life and their families as well as the well-being of health professionals.

Accordingly, four hypotheses (H1-H4) were specified:

H1: We assume that people who dehumanize the hospitalized patient will self-dehumanize.

H2: We assume that autonomous people will not dehumanize and self-dehumanize.

H3: We assume that people with insecure attachment will dehumanize the hospitalized patient.

H4: We assume that people with insecure attachment will not self-dehumanize.

## Materials and methods

Sampling

Convenience sampling was used to select the participants in the study. People employed in health care were drawn from two large hospitals of Athens, a General Hospital (Sotiria) and the Dromokaitio Psychiatric Hospital of Attica. Of these, 135 were mental health professionals (doctors, nurses and psychologists working in psychiatric departments; 20 from the General Hospital and 115 from Dromokaitio) and 134 were staff in other departments of the General Hospital (doctors and nurses working in the pathology, surgery and intensive care units of the hospital). These participants received the questionnaires following a personal approach by the researcher to the nursing departments where they worked. For the selection of a sample of the general population, individuals living in Athens were approached at work and at their place of residence: 84 completed the questionnaires. Ten questionnaires were canceled because participants had not completed all of the questionnaires. The total sample for analysis consisted of 353 people.

The study was conducted in the period June 2016-December 2016. The ethics committee number of approval from the General Hospital Sotiria was 2016-10123. Questionnaires were self-completed, by the health professionals at their workplace and by the general population in their homes. The information provided to participants was that the research concerned the hospitalized patient. No further information was provided to avoid bias. They were also informed that the investigation was voluntary and anonymous and there were no right or wrong answers. The average time to complete the questionnaire was 15 minutes.

Measures

In addition to basic demographic items (gender, age, educational level), participants were asked to complete a set of questionnaires with established psychometric properties, as follows.

Dehumanization Questionnaire

The questionnaire, which is based on the model of Haslam [1], has two dimensions: mechanistic and animalistic dehumanization. The lower the scale score, the greater the dehumanization, and the higher the score, the greater humanity. This is a questionnaire consisting of eight pairs of characteristics (eg distant-cold, with warmth), which must be attributed to the hospitalized patient. Statements 1, 2, 3, 4 (eg distant/cold, with warmth) constitute mechanistic dehumanization and statements 5, 6, 7, 8 (eg instinctive/rational) constitute animalistic dehumanization. From the subscale of mechanistic dehumanization, statement 2 is coded in reverse. The scoring is done according to a 9-point Likert scale where 1 corresponds to the attribute on the left side of the scale (eg without autonomy and will) and 9 to the attribute on the right side of the scale (autonomous and voluntary). The maximum score that can be given per dimension is 36. The questionnaire has been translated and adapted into Greek by Sakalaki et al. [11,12]. The internal consistency of the overall questionnaire, in this study, was Cronbach’s α = .77. For the mechanistic subscale, α = .66, and for the animalistic subscale α = .76. The questionnaire is provided in Appendix A.

Mechanistic Self-Dehumanization Scale

The questionnaire consists of 14 items and measures the performance of human nature characteristics in the self. The higher the value the greater the self-dehumanization. The internal consistency of the scale, in this study, was Cronbach’s α = .78. The Mechanistic Dehumanization Scale [11] is inspired by the model of Haslam [1] and Gray et al. [7]. The rating is done with a 9-point Likert scale (1 = "strongly disagree", 9 = "strongly agree") where the degree of agreement described in each statement is stated. The questionnaire is provided in Appendix B.

Human Nature (HN) and Human Uniqueness (HU) Characteristics Questionnaire

The questionnaire consists of eight items regarding the attribution of HN, HU characteristics in self. The questionnaire includes two subscales that assess the Human Nature and Human Uniqueness dimensions. The rating is done according to a 9-point Likert scale (1 = "not at all", 9 = "too much"), which indicates the degree of agreement of the situation described in each statement. The maximum score that can be given per factor is 36. The higher the value, the higher the attribution of the HN and HU in itself. Ιnternal consistency, in this study, was Cronbach’s α = .82 for Human Nature and α = .67 for Human Uniqueness [13]. The questionnaire is provided in Appendix C.

General Causality Orientation Scale

This questionnaire consists of 12 vignettes. In each vignette, three alternative answers are suggested, corresponding to the autonomous, controlled and impersonal orientations. The higher the scale score, the greater the performance in the factor. The rating is done with a 7-point Likert scale (1 = "not at all likely", 7 = "very likely") where the degree of agreement of the situation described in each statement is stated. The maximum score that can be given per factor is 84. The questionnaire has been translated and adapted into Greek by Sakalaki and Fousiani [14]. The internal consistency of the overall scale was Cronbach's α = .75, in this study and for each orientation it was: autonomous orientation α = .75, controlled orientation α = .74, impersonal orientation α = .77 [15]. The questionnaire is provided in Appendix D.

Adult Attachment Questionnaire

The questionnaire consists of 36 items and includes two subscales with 18 items each, which assess the two dimensions (stress/obsession, avoidance) of adult attachment [16]. Respondent states whether he/she agrees with each of the 36 sentences on a 7-point Likert scale (1 = "strongly disagree", 7 = "I totally agree"). Single-sentence sentences are the subscale of avoidance. Of these, 3, 7, 15, 17, 19, 21, 23, 25, 29, 31, 33, 35, have reverse coding. Sentences in even numbers are the subscale of stress/obsession. Of these, 18 and 22 have reverse coding. The average in each sub-scale gives the score of the attachment in the two dimensions, and the low values ​​of avoidance refer to safety [16,17]. The questionnaire is distinguished by satisfactory reliability and internal consistency. Cronbach’s α of the questionnaire is .92, for the avoidance subscale .91 and for the stress/obsession subscale .90, in this study. The questionnaire is provided in Appendix E.

Statistical analysis

The investigation was performed with a series of analyzes. Statistical Package for Social Sciences (SPSS) Base and SPSS Advanced Models (Edition 25, 2018; IBM Corp., Armonk, NY, USA) were used for analyzes. The analyzes that were performed were one-way ANOVA, regression, two-way ANOVA and effect size. Also, post hoc multiple comparison was performed using the Bonferroni criterion.

## Results

Of the participants, 34% were 18-35 years old, 42% were 36-45 years old, 24% were 46-60 years old and the rest were over 60 years old, 21% are high school graduates, 50% Bachelor graduates and 28% held a postgraduate degree. Of the participants, 103 were men and 250 were women (Table 1).

**Table 1:** Demographic characteristics (N=353)

Variable		Ν	%
Gender			
Men		103	29.2
Women		250	70.8
Age			
18-35		120	34
36-45		148	41.9
45-over 60		85	24.1
Educational level			
Secondary school		78	21.1
Bachelor’s degree		178	50.4
MSc-PhD		97	27.5
Sample			
Mental health professionals		135	38.2
Health professionals		134	38
General population		84	23.8

As shown, there was a statistically significant difference (p = .005 in one-way ANOVA) only in terms of Mechanistic Dehumanization between general hospital staff, the general population and mental health professionals (Table 2). Cohen’s index η2 = .09 shows a large effect size.

**Table 2:** Differentiation between Mental Health Professionals, Health Professionals and the General Population on the variables Mechanistic Dehumanization and Animalistic Dehumanization (one-way ANOVA).

Dehumanization	Sample	N	M	SD	F	η^2^	p
Mechanistic	Mental health professionals	135	5.0	1.0	F(2,350) = 3.29	.09	.038
	Health professionals	134	5.1	1.0			
	General population	84	4.7	1.0			
	Total	353	5.0	1.1			
Animalistic	Mental health professionals	135	5.0	1.3	F(2,350) = .165	.09	.850
	Health professionals	134	5.1	1.3			
	General population	84	5.1	1.2			
	Total	353	5.1	1.3			

In order to identify between which groups there is a significant difference, a post hoc multiple comparison was performed using the Bonferroni criterion. According to the results of the analysis, the health professionals (M = 5.1, SD = 1) showed significantly greater Mechanistic Dehumanization compared to the general population (M = 4.7, SD = 1). There was no statistically significant difference between the other groups. It is also observed from Table 2 that there was no statistically significant difference (p = .85) in terms of animalistic dehumanization between general hospital staff, mental health professionals and the general population. No associations of self-determination were observed with the animalistic and mechanistic dehumanization of the hospitalized patient, in the regression (p > .05). Regarding mechanistic self-dehumanization, there was a statistically significant difference F (2, 350) = 5.5, p = .004, η2 = .20) between mental health professionals and health professionals. According to the results of the analysis, the general population (M = 4, SD = .9) self-dehumanized significantly more than mental health professionals (M = 3.5, SD = .9).

There was no statistically significant difference between health professionals and the general population (p > .05). It seems that the general population self-dehumanizes more mechanically than mental health professionals.

From the attachment dimensions of the examined groups, avoidance was significantly positively associated with the animalistic dehumanization of patients (β = .23, t = 2.97, p = .003, f2 = .02), Cohen’s index f2 = .02 indicating small effect size. Anxiety/obsession was not correlated with the animalistic dehumanization of patients (p > .05). From the attachment dimensions of the examined groups, avoidance was significantly and positively associated with the mechanistic dehumanization of patients (β = .20, t = 3.17, p = .002, f2 = .03), Cohen’s index f2 = .03 indicating small effect size. Anxiety/obsession was not associated with mechanistic dehumanization of patients (p > .05).

The scales of uniquely human characteristics and mechanistic self-dehumanization were used to examine the self-dehumanization of the three groups. It appears that there is a significant difference in terms of the characteristics of Human Nature (HN), F (2, 350) = 5.47, p = .005, as well as in the Human Uniqueness (HU) characteristics, F (2, 350) = 5.40, p = .005, to themselves (mental health professionals, health professionals and the general population) in their daily lives (Table 3). Cohen’s index η2 = .12 for the human nature characteristic indicates medium effect size, while for the human uniqueness characteristics Cohen’s η2 = .06, which indicates small effect size.

**Table 3:** Differentiation between Mental Health Professionals, Health Professionals and the General Population on the variables Characteristics of Human Nature and Uniquely Human

Variables	Sample	N	M	SD	F	η^2^	p
Human nature	Mental health professionals	135	7	1.2	F(2,350)=5.47	.12	.005
	Health professionals	134	6.6	1.1			
	General population	84	6.6	1.1			
	Total	353	6.8	1.2			
Human uniqueness	Mental health professionals	135	7.4	1.1	F(2,350)=5.4	.06	.005
	Health professionals	134	7.4	1			
	General population	84	6.9	1.1			
	Total	353	7.3	1.1			

Table 3 shows that mental health professionals and the rest of the health professionals considered themselves more human than the general population.

The self-determination (autonomy, control, impersonal orientation) of the mental health professionals, health professionals and the general population was associated with their mechanistic self-dehumanization. It was found that the autonomy orientation was associated significantly negatively (β = -.33, t = -4.97, p < .001), and impersonal orientation significantly positively (β = .36, t = 6.75, p < .001), with their mechanistic self-dehumanization. Control orientation did not have a significant association (p > .05). Cohen’s index f2 = .26 indicates average effect size. From the attachment dimensions of the examined groups, both avoidance (β = -.32, t = -6.65, p < .001) and stress/obsession had significant negative associations with mechanistic self-dehumanization (β = -.14, t = -2.92, p = .004). Cohen’s index f2 = .28 indicates average effect size.

Gender was not significantly associated with the mechanistic and animalistic dehumanization of patients, the attribution of Human Nature and Human Uniqueness characteristics to the self of the examined groups and their mechanistic self-dehumanization. Also, age did not have a statistically significant association with the mechanistic and animalistic dehumanization of patients. However, age did have a significant association with the attribution of Human Nature and Human Uniqueness characteristics on themselves and on their mechanistic self-dehumanization. Furthermore, educational level had a statistically significant association with the mechanistic and animalistic dehumanization of patients, on the attribution of the Human Uniqueness characteristics on the self and on mechanistic self-dehumanization.

## Discussion

The aim of the research was to study the dehumanization of the hospitalized patient by mental health professionals, other health professionals and the general population, as well as to study self-dehumanization of mental health professionals, other health professionals and the general population.

The findings show that the hospitalized patient is dehumanized more mechanistically by health professionals (doctors and nurses working in the pathology and surgery clinics of the hospital) than by the general population. This finding is compatible with the literature, showing that the patient's subjective experience is neglected in favor of objective and technologically mediated information and emphasis is placed on interventions performed on a passive individual whose agency and autonomy are absent [1,18]. Doctors tend to see their patients as inactive bodies [19] and as mechanical systems with interacting parts [18] and according to this view, the disease can be explained as motor dysfunction [20]. Importantly, health care workers also show a reduced attribution of humanness to patients [21,22]. This dehumanization is seen as a protective coping strategy for staff, as dehumanizing patients is associated with reduced emotional involvement and reduced risk of burnout [22]. The self-determination of mental health professionals, health professionals and the general population does not affect the dehumanization of the hospitalized patient. The above finding is compatible with findings of similar research [12].

From the attachment dimensions (anxiety/obsession, avoidance), it appears in the present study that avoidance has a positive effect on both the mechanistic and the abnormal dehumanization of the hospitalized patient by the mental health specialists, other health professionals and the general population. Avoidance reflects the degree to which a person distrusts the goodwill of another and tries to maintain their independence and emotional distance from the other [23,24]; it is characterized by negative representations of others, forced self-confidence and preference for emotional distance [25-28]. Individuals with high avoidance find it difficult to "read" the minds of others as well as to understand the behaviors of others as important and predictable [29]. Denial of the mind to the other is the essence of dehumanization [30].

The above finding is very important in clinical practice because avoidance is not consistent with empathy, which is a structural feature in the therapeutic relationship between specialist and patient, contributes to compliance and implementation of medical instructions, and is related to the effectiveness of treatment, when the patient has a physician who treats him or her humanely [20]. At the same time, the reduction of empathy probably contributes directly to the increase of dehumanization, as suggested by Haslam [1].

It seems that the general population self-dehumanizes more mechanically than mental health professionals. Mental health professionals who work in psychiatric clinics are more trained in communication skills than the general population, may have to deal with patients who attend and are treated against their will on a prosecutor's order and have to deal with families of patients who are possessed by ambivalence and guilt regarding the prosecutor's order. All of the above contribute to mental resilience and promote the need for social connection, which leads to greater humanity of the self [13].

Research findings also show that autonomous people (mental health professionals, health professionals and the general population) attribute the characteristics of Human Nature and Human Uniqueness to themselves. In other words, autonomous orientation makes individuals perceive themselves as more human. In addition, autonomous orientation is negatively correlated with self-humanization [12]. The above findings confirm that a greater degree of autonomy is associated with the experience of feeling more of a human and less of a machine [31]. On the other hand, impersonal orientation (mental health specialists, health specialists and the general population) is negatively related to the attribution of the characteristics of Human Nature and Human Uniqueness to oneself and positively to self-dehumanization, because the individual believes that achieving the desired results is beyond their control and depends to a large extent on fate or luck [32]. In this case the internal motivations are undermined and the person has a sense of helplessness and lack of motivation [15].

Impersonally oriented individuals have an external seat of control over behaviors and reinforcements [15], which leads to their self-humanization. Finally, the controlled orientation (mental health experts, health experts and general population) does not significantly predict self-dehumanization, neither is it statistically significantly correlated with it, nor with the attribution of Human Nature and Human Uniqueness characteristics to itself. These findings contradict the research of Moller and Deci [31], according to which controlled orientation is positively correlated with dehumanization and leads individuals to dehumanize themselves. It is possible that people who regulate their behavior based on control from others or from themselves (self-control) do not perceive control as something negative or do not realize that they are not acting autonomously. As a result, they do not perceive themselves as an object directed by the instructions of others or by an internalized control.

In addition, the research findings of the present study show that insecure bonding does not significantly affect self-dehumanization, possibly because people who avoid high self-avoidance are characterized by positive representations of themselves and negative representations of others [25-28], whenever they do not self-humanize and attribute the characteristics of humanity to themselves. However, people with high stress are characterized by negative representations of themselves and positive representations of others [25,26,33,28].

It seems that the gender of mental health professionals, health professionals and the general population does not affect the dehumanization of the hospitalized patient or the dehumanization of mental health professionals, health professionals and the general population. This finding contradicts research findings that argue that women self-humanize more than men because they are objectified [34,35].

Regarding the age of mental health professionals, health professionals and the general population, it appears from the findings of the present study that people aged 46-60 years mechanically dehumanize the hospitalized patient more than people over 60, possibly due to existential concerns and mortality [35], as this age group is close to retirement, has experienced losses and is facing a new phase of life. The above age groups do not appear to be self-dehumanizing in the present study, although according to the findings in social exile, groups that dehumanize out-groups simultaneously self-dehumanize [13].

Finally, the findings of the present study show that the educational level of mental health professionals, health professionals and the general population plays a role in both the dehumanization of the hospitalized patient and the dehumanization of the self. It seems that mental health professionals who hold a postgraduate/doctoral degree and graduates of higher education mechanistically dehumanize the patient more than mental health professionals who are high school graduates. Dehumanization is also a central process in prejudice and stigma [36] and increases the social rejection of the mentally ill. Meta-analysis results show that the social rejection of the mentally ill has remained alarmingly stable over the last 20 years [37]. Indeed, health service users often rate mental health personnel as one of the groups that stigmatize the mentally ill [38]. Discrimination provokes more negative emotions in both the public and health professionals [39]. Also, university graduates and postgraduate degree holders usually hold positions of responsibility and are called upon to make decisions for patients whose autonomy and individuality are affected because of involuntary hospitalization [40,41] and their dehumanization is used as a defense against stress [22] and to deal with difficult human situations without burnout [22,42].

Naturally, our research also faced some limitations. First, women outnumbered men in the sample, and for this reason, the future line of research is proposed to focus on exploring the existence of non-gender differences associated with changes in the levels of dehumanization of others and self-dehumanization. Second, the samples collected in the studies of this dissertation, although sufficiently large for statistical analysis, were not obtained by random sampling. Third, the present study did not include any staff working in the hospital laboratory. Future research could cover these areas, as these specialties do not have direct contact with persons but biological materials and depictions of hospitalized persons.

## Conclusions

From the results it seems that there is a mechanistic dehumanization of the hospitalized patient by the health professionals, therefore it is necessary to take measures to prevent the phenomenon. Also, the research findings show that the insecure attachment protects against self-dehumanization, as it acts as a defense for self-dehumanization and therefore there is a need to take measures that will contribute to the creation of secure relationships that are important for the therapeutic context. Finally, it is necessary to take measures that will enhance the autonomy of health professionals, as the findings show that autonomous people neither dehumanized nor self-dehumanized. 

## Appendices

Appendix A

To what extent do you think your patients have the following characteristics? 1 approaches the attribute on the left side of the nine-point scale and 9 approaches the attribute on the right side of the nine-point scale.

1. Distant/Cold  1  2  3  4  5  6  7  8  9  With Warmth

2. Widely Spirit  1  2  3  4  5  6  7  8  9  Without breadth spirit

3.Does not respond emotionally to others  1  2  3  4  5  6  7  8  9  Responds emotionally to the others

4. Without autonomy and will  1  2  3  4  5  6  7  8  9  Autonomous and Voluntary

5. Instinctive   1  2  3  4  5  6  7  8  9  Rational 

6. Uncivilized   1  2  3  4  5  6  7  8  9  Civilized

7. Childish    1  2  3  4  5  6  7  8  9  Mature

8. Peasant    1  2  3  4  5  6  7  8  9 Sophisticated

Appendix B

Note the extent to which you agree or disagree with the following suggestions

1. I usually do not work emotionally

Disagree Absolutely 1  2  3  4  5  6  7  8  9  Strongly Agree

2. When I have to make choices, I trust my values and beliefs.

Disagree Absolutely 1  2  3  4  5  6  7  8  9  Strongly Agree

3. I have the impression that my actions do not come from within me, but that they are dictated to me by things, circumstances or others.

Disagree Absolutely 1  2  3  4  5  6  7  8  9  Strongly Agree

4. My actions stem from my own motives, from what I want or from what I am interested in doing.

Disagree Absolutely 1  2  3  4  5  6  7  8  9  Strongly Agree

5. I am not open to stimuli from the outside world that are foreign or unknown to me.

Disagree Absolutely 1  2  3  4  5  6  7  8  9  Strongly Agree

6. In my relationships with others I like to express my feelings.

Disagree Absolutely 1  2  3  4  5  6  7  8  9  Strongly Agree

7. I often feel that I lack depth, that I am a little superficial.

Disagree Absolutely 1  2  3  4  5  6  7  8  9  Strongly Agree

8. I feel warmth in my relationships with others.

Disagree Absolutely 1  2  3  4  5  6  7  8  9  Strongly Agree

9. I am receptive to any new knowledge. It makes me curious.

Disagree Absolutely 1  2  3  4  5  6  7  8  9  Strongly Agree

10. I believe that most of my actions and choices in life come from my own autonomous intentions and preferences.

Disagree Absolutely 1  2  3  4  5  6  7  8  9  Strongly Agree

11. I usually respond to the feelings of others.

Disagree Absolutely 1  2  3  4  5  6  7  8  9  Strongly Agree

12. I am open to any new experience.

Disagree Absolutely 1  2  3  4  5  6  7  8  9  Strongly Agree

13. Sometimes I behave mechanically, without thinking about what I do.

Disagree Absolutely 1  2  3  4  5  6  7  8  9  Strongly Agree

14. People sometimes work with automation. Under certain conditions, a part of their mind works mechanically or their actions are done without having thought or chosen for themselves. How often would you say that you have had this experience?

Never  1  2  3  4  5  6  7  8  9  Often

Appendix C

Below are 8 suggestions, please note the degree to which you feel this way in your daily life.

1. I feel like I'm open-minded and able to think things through.

Not at all  1  2  3  4  5  6  7  8  9  Very much

2. I feel like I am emotional and can respond to the feelings of others and be warm.

Not at all  1  2  3  4  5  6  7  8  9  Very much

3. I feel like I am passive, like I lack energy.

Not at all  1  2  3  4  5  6  7  8  9  Very much

4. I feel like I'm an engineer and cold, like a robot.

Not at all  1  2  3  4  5  6  7  8  9  Very much

5 I feel rational and rational as if I were smart.

Not at all  1  2  3  4  5  6  7  8  9  Very much

6. I feel like I am refined and cultured.

Not at all  1  2  3  4  5  6  7  8  9  Very much

7. I feel like I do not have self-control, like animals.

Not at all  1  2  3  4  5  6  7  8  9  Very much

8. I feel like I'm uncultured

Not at all  1  2  3  4  5  6  7  8  9  Very much

Appendix D

The following sentences refer to a series of hypothetical situations. Each sentence describes an event with three possible ways of reacting to it. Please read all the sentences and imagine that each of the hypothetical situations actually happens to you. Then think about the extent to which the possible answers match the way you would react to each situation. Depending on how likely you are to react in the way described in each answer, choose the corresponding number from 1 to 7, where 1 indicates a low probability and 7 a high probability.

1. You are offered a new position in a company where you have worked for some time. The first thing that probably bothers you is: a) What will happen if I can not cope with my new duties?

Not at all possible 1  2  3  4  5  6  7  Very likely b) Will I do more things through this position?

Not at all possible 1  2  3  4  5  6  7  Very likely c) I wonder if my new job will be interesting.

Not at all possible 1  2  3  4  5  6  7  Very likely

2. You have a daughter who goes to school. On Parents' Day, the teacher tells you that your daughter is not doing well and that she does not seem to be reading. You: a) Discuss it with your daughter to better understand what the problem is.

Not at all possible 1  2  3  4  5  6  7  Very likely b) You scold her and hope that in the future she will do better.

Not at all possible 1  2  3  4  5  6  7  Very likely c) You try to make sure it changes, because it needs to read more.

Not at all possible 1  2  3  4  5  6  7  Very likely

 3. A few weeks ago you had a professional interview. You will receive a letter from the post office informing you that the post has been filled by someone else. What you will think is: a) It does not matter what you know, but who you know.

Not at all possible 1  2  3  4  5  6  7  Very likely b) I was probably not good enough for this job.

Not at all possible 1  2  3  4  5  6  7  Very likely c) For some reason they did not see that my qualifications fit their needs.

Not at all possible 1  2  3  4  5  6  7  Very likely

4. You are a facility supervisor and you have to give a coffee break to three employees, but they cannot all be absent together. You handle it as follows: a) Explain the problem to the three employees and ask them to discuss the break schedule with you.

Not at all possible 1  2  3  4  5  6  7  Very likely b) You just set the time that everyone can take a break, thus avoiding any problems.

Not at all possible 1  2  3  4  5  6  7  Very likely c) You ask a senior what to do or what had happened in the past in a similar case.

Not at all possible 1  2  3  4  5  6  7  Very likely

5. A close friend (of the same sex as you) has been in a bad mood lately and sometimes gets angry at you for "nothing." You: a) You tell him what you have noticed in his behavior and you try to find out what is happening to him.

Not at all possible 1  2  3  4  5  6  7  Very likely b) You ignore it because there is nothing you can do about it.

Not at all possible 1  2  3  4  5  6  7  Very likely c) You tell him that you are willing to spend time with him only if he tries harder to control himself.

Not at all possible 1  2  3  4  5  6  7  Very likely

6. You just received the results of a test you did, and you find that you did not do very well. Your initial reaction is probably: a) "I can not do anything right" and you feel sad.

Not at all possible 1  2  3  4  5  6  7  Very likely b) "I wonder how I could have done so badly" and you feel frustrated.

Not at all possible 1  2  3  4  5  6  7  Very likely c) "This stupid test shows nothing" and you feel angry.

Not at all possible 1  2  3  4  5  6  7  Very likely

7. You have been invited to a big party where you know very few people. As you look forward to the night, you think: a) You will try to participate in everything that happens in order to have a good time and not show that you feel bad.

Not at all possible 1  2  3  4  5  6  7  Very likely b) You will find some people with whom you can hang out.

Not at all possible 1  2  3  4  5  6  7  Very likely c) You will probably feel somewhat isolated and unnoticed.

Not at all possible 1  2  3  4  5  6  7  Very likely

8. You are invited to organize a picnic for you and your close associates. The way you will do it could be as follows: a) You will take it all on your own: that is, you will make the most important decisions on your own.

Not at all possible 1  2  3  4  5  6  7  Very likely b) You will imitate the previous ones: you can not design it, therefore you will do it following the way it was done the previous time.

Not at all possible 1  2  3  4  5  6  7  Very likely c) You will seek the participation of others: you will get ideas from others before you make the final plans.

Not at all possible 1  2  3  4  5  6  7  Very likely

9. You recently opened a position in your workplace that could mean promotion for you. However, this position was offered to a colleague instead of you. Assessing the situation, you might think that: a) You did not expect to take the position anyway because you are usually rejected.

Not at all possible 1  2  3  4  5  6  7  Very likely b) Your colleague probably made the "right" moves to take the position.

Not at all possible 1  2  3  4  5  6  7  Very likely c) You would try to identify the factors that led to your rejection.

Not at all possible 1  2  3  4  5  6  7  Very likely

10. You are starting a new professional career. What concerns you the most is: a) If you can do this job without getting too tired.

Not at all possible 1  2  3  4  5  6  7  Very likely b) Whether you are interested in such work.

Not at all possible 1  2  3  4  5  6  7  Very likely c) If there are good prospects for development.

Not at all possible 1  2  3  4  5  6  7  Very likely

11. A woman who works for you does her job well in general. However, in the past two weeks her performance has not been so good and she shows less interest. Your most likely reaction is: a) You tell her that her performance is worse than you expected and that she needs to start working harder.

Not at all possible 1  2  3  4  5  6  7  Very likely b) You talk to her about this problem and inform her that you want to help her overcome it.

Not at all possible 1  2  3  4  5  6  7  Very likely c) You can not know how to make it come back.

Not at all possible 1  2  3  4  5  6  7  Very likely

12. The company you work for promotes you to a position in a city far from your current home. As you consider this move you are likely to: a) You are interested in the new challenge and at the same time you feel a little nervous.

Not at all possible 1  2  3  4  5  6  7  Very likely b) You feel excited for the highest position and the salary she deserves.

Not at all possible 1  2  3  4  5  6  7  Very likely c) You feel stressed and anxious about the changes that will occur

Not at all possible 1  2  3  4  5  6  7  Very likely

Appendix E

The following 36 suggestions are about how you feel in love relationships / relationships, including marriage. We are interested in how you experience these relationships in general and not just what happens in a current relationship. Note next to each sentence whether you agree or disagree with it based on the following scale:

Strongly agree  1  2  3  4  5  6  7  Strongly disagree

1 --- I prefer not to show my partner how I feel deep down. 2__ I am afraid that I will lose the love of my partner. 3__ I feel comfortable sharing personal thoughts and feelings with my partner. 4__ I am often worried about the idea that my partner will not want to stay with me 5__ I find it difficult to allow myself to rely on sexual partners. 6__ I am often worried about the idea that my partner does not really love me. 7__ I feel very comfortable being emotionally close with sexual partners. 8__ I'm worried that my sexual partners will not care about me as much as I do about them. 9__ I do not feel comfortable 'opening up' to sexual partners. 10__ I often wish my partner's feelings for me were as strong as mine for him / her 11__ I prefer not to be emotionally very close to sexual partners. 12__ I am very worried about my relationships. 13__ I feel uncomfortable when my sexual partner wants to be emotionally very close to me. 14__ When my partner is away from me, I worry that he might be interested in someone else. 15__ I find it relatively easy to get emotionally close to my partner. 16__ When I show my feelings to my sexual partners, I am afraid that they will not they feel the same for me. 17__ It is not difficult for me to get emotionally close to my partner. 18__ I rarely worry that my partner may leave me. 19__ I usually discuss my problems and worries with my partner. 20__ My love partner makes me question myself. 21__ Helps me to turn to my partner in times of need. 22_ I rarely worry about the idea that I may be abandoned 23__ I tell my partner almost everything. 24__ I find that my partners do not want to come to me emotionally as much as I would like. 25__ I discuss the things that concern me with my partner 26__ Sometimes my sexual partners change their feelings for me for no apparent reason 27__ I feel nervous when a partner comes too close to me emotionally 28__ My desire to be emotionally very close to others sometimes frightens them and drives them away. 29 .__ I feel comfortable relying on my love partner. 30 .__ I'm afraid that once a love partner knows me well, he / she will not like who I really am. 31__ I find it easy to rely on sexual partners. 32__ I'm angry that I do not get the affection and support I need from my partner. 33 .__ It is easy for me to be tender with my love partner. 34 .__ I'm worried that I'm lagging behind other people. 35__ The partner who really understands me and my needs. 36 .__ It seems to me that my partner only pays attention to me when I am angry.
